# Supplementary material for: Willingness and ability to pay for healthcare insurance: A cross-sectional study of Seven Communities in East and West Africa (SevenCEWA)
Source: PLOS Glob Public Health. 2021 Nov 24;1(11):e0000057. doi: 10.1371/journal.pgph.0000057 (PMC10021733; doi:10.1371/journal.pgph.0000057)
Supplement: Attachment — Submitted filename: rebuttal_letter.doc [file pgph.0000057.s001.doc]

**Rebuttal Letter: Response to the reviewers’ comments**

Title: Willingness and ability to pay for healthcare insurance: a cross-sectional study of Seven Communities in East and West Africa (SevenCEWA)

Dear Editor,

Thank you for giving us the opportunity to give response and make corrections to the comments and advice made by the 2 reviewers to improve our manuscript.

Kindly find below the point-to-point responses to these comments.

Best regards

Dr. Bolarinwa Oladimeji Akeem

*Corresponding Author*

| **Reviewer Number** | **Original comments of the reviewer** | **Reply by the author(s)** | **Changes done on the line number** |
| --- | --- | --- | --- |
| 1 | Overall, it is a good study and manuscript. However, contingency valuation method used in measuring WTP in the study was not clearly explained in the methodology section and not presented in the result section | Mixed contingency methods were used in this study. For all study sites with low insurance coverage a simple open-ended question was asked. In Kenya Viwandani with highest insurance coverage, a bidding method was asked to explore the preferences for different insurance packages available. The maximum amount willing to pay were aggregated and used across the sites. Therefore, the bidding pattern was not shown in the results. Instead, we presented median amount willing to pay for easy comparison across the sites and countries. | 161 - 173  176 - 178 |
| 2 | Some minor revisions are required. Author’s attention has been drawn to sentences that need to be reviewed |  |  |
| 2 | Line 68 to 71 “Retired respondents …………..health insurance scheme.” | Corrected | 68 - 69 |
| 2 | Line 86 to 89 “Whereas only a few ……..African Governments.” | Corrected | 91 - 92 |
| 2 | Line 89 to 90 “In addition, …………… lagging for their population.” | Rephrased | 94 - 96 |
| 2 | Line 93 “For a success….. generation is cardinal” (‘successful’ for ‘success’ | Corrected | 100 |
| 2 | Line 118 to 120 | No correction stated and no grammar error spotted by the authors |  |
| 2 | Line 151 “………while ATP for healthcare………” | Corrected | 161 |
| 2 | Line 257 to 260 “This is similar to …………scheme in Ethiopia.” | Punctuation corrected | 277 |
| 2 | Line 274 “……pose impractical challenges to being ……” | Rephrased | 296 - 297 |
| 2 | Line 334 “……securely stored in in both hard….” | Corrected | 359 |
| 2 | This is an interesting paper for LMICs working at establishing some form of health insurance to attain UHC. However, payment of premiums is not the only way to go. I find that the paper does not come out strongly on equity and social justice, that which UHC and SDG 3 partly aims at. The crux of the paper appears to be “… if people are willing to pay and have the ability to pay some amount as premium, they can enrol on a social health insurance scheme towards the attainment of UHC and SDG3”. “And so, it will be good that policy makers consider this.” [If this is not what the authors are saying, then it is not very clear to me]. In practice, it does not work that way (policy implementation is not that linear). What happens to those who cannot afford these premiums? What are the reasons why some people are not willing to pay or enrol onto insurance schemes as found in the results? I find the discussion of the findings (mainly) and introduction, a bit deficient in this regard. | We found this comment very valuable to the policy directions for the findings from our study. Beyond the WTP and ATP to pay for health insurance, the issues of equity and social justice is key for the attainment of UHC and SDG3 goal in Africa. We have therefore incorporated this perspective into the manuscript.    We therefore, introduce studies that have reported equity and social injustice issues in some established social health insurance in Africa in the introduction section. We further discussed and concluded on these. | 74 - 76  129 -131  290 - 296  349 - 352 |
| Editor |  | References rearranged | 125 |
| Editor |  | Reference list changed | 367 - 453 |
|  |  |  |  |
